# Seneca valley virus VP4 protein regulates the transcription of different cytokines *in vitro*

**DOI:** 10.3389/fvets.2025.1675546

**Published:** 2025-10-16

**Authors:** Chaoliang Leng, Yu Ge, Mengfan Ruan, Wenxiao Zhao, Ximei Yang, Sainan Gao, Hongyue Zhai, Dandan Li, Dan Rao, Jianguo Dong

**Affiliations:** ^1^Henan Key Laboratory of Insect Biology, Henan Provincial Engineering and Technology Center of Animal Disease Diagnosis and Integrated Control, Henan Province Engineering Research Center of Insect Bioreactor, China-UK International Joint Laboratory for Insect Biology of Henan Province, Nanyang Normal University, Nanyang, China; ^2^School of Animal Husbandry and Veterinary Medicine Engineering, Xinyang Agriculture and Forestry University, Xinyang, China; ^3^AnLing Biopharmaceutical (Suzhou) Co., Ltd, Suzhou, China

**Keywords:** Seneca valley virus, VP4 protein, eukaryotic expression, interleukin, chemokine, tumornecrosis factor

## Abstract

To understand the effect of Seneca valley virus (SVV) VP4 protein on innate immune factors, the VP4 gene was cloned into the pEGFP-C1 expression plasmid to construct the pEGFP-C1-VP4 recombinant plasmid. After the recombinant plasmid was transfected into 293 T cells, the cell fluid was collected 24 h after transfection for western blot assay to identify the correctness of VP4 protein expression. Cell culture medium was collected from un-transfected and transfected cells at three time points (12, 24, and 36 h). mRNA expression levels of cytokines (IL-1*α*, IL-1β, CCL-2, CCL-5, CXCL-10, and TNF-α) at three time points were detected by quantitative real-time PCR (qPCR) method, and relative quantitative analysis was performed by 2-ΔΔCt method. The results indicated that the expressed SVV VP4 protein exhibits good activity *in vitro*. Overexpression of the VP4 protein could significantly promote the transcription of IL-1α and IL-1β at 24 and 36 h. In addition, the transcription of CCL-2 and CCL-5 was also significantly promoted at 36 h, whereas the transcription of CCL-10 was significantly promoted only at 12 h. The TNF-*α* transcription was significantly inhibited at all the three time points. This study provides an important basis for the pathogenic mechanism of SVV and vaccine design in the future.

## 1 Introduction

Seneca valley virus (SVV), also known as seneca type A virus (SVA), belongs to the *Picornaviridae* family and the *Senecavirus* genus (1). The virus particle is icosahedral symmetrical no envelope and the genome is single-stranded positive-strand RNA. After SVV infecting pigs, it can cause obvious blisters and ulceration lesions on the nasal mirror and hooves of pigs, and cause symptoms such as lameness, loss of appetite, drowsiness and fever in pigs at the same time. Since its clinical symptoms are highly similar to those of foot-and-mouth disease (FMD), swine vesicular disease (SVD), and vesicular stomatitis (VS), it is difficult to distinguish between them, which increases the difficulty of its prevention (2). In 2015, SVV broke out in China. Subsequently, SVV outbreaks occurred successively in Fujian, Guangdong, Heilongjiang and other places (3–6). Retrospective monitoring showed that there were varying degrees of SVV contamination in pig herds in seven provinces (autonomous regions), namely Hunan, Yunnan, Xinjiang, Liaoning, Fujian, Hubei, and Guangxi, from 2016 to 2018 (7). Since 2015, the Chinese SVV isolates have undergone rapid evolution and been clustered into five genetic branches (8). Currently, SVV still widely exists in various countries and poses potential hazards to pig farms (9).

Among the SVV structural proteins, VP1, VP2, and VP3 are all exposed on the surface of the viral particle, while the VP4 protein, with a length of approximately 72 amino acids, is located inside the viral capsid and is relatively conserved among the four structural proteins (10). Mutations in VP4 can alter the permeability of the host cell membrane and enhance the ability of the viral genome to be delivered to cells. The VP4 protein could induce the production of neutralizing antibodies during virus infection of host cells (11). Moreover, Panjwani et al. found that during the process of small ribonucleic acid viruses entering cells, the internal capsid protein VP4 is released from the virus and interacts with the cell membrane (12). These results indicate that the VP4 protein plays an important role in promoting the membrane fusion process between the virus and the host cell.

Cytokines play a significant role in resisting viral invasion (13). By exploring the changes of cytokines after viral infection, identifying key cytokines that inhibit viral proliferation, and clarifying their mechanisms of action, it is helpful to develop protein products for treatment or as vaccine adjuvants for the treatment and prevention and control of diseases. However, there are still no relevant studies on the regulation of cytokines by SVV VP4 at present. To explore the effect of SVV VP4 protein on cytokines, in this study, the constructed pEGFP-C1-VP4 plasmid was transfected into 293 cells. The VP4 protein was identified as having *in vitro* activity by western blotting. Subsequently, the cells were recovered and nucleic acids were extracted at 12, 24, and 36 h. The transcriptional levels of interleukin (IL) (IL-1*α* and IL-1β), chemokines (CCL-2, CCL-5, and CXCL-10) and tumor necrosis factor (TNF-α) were detected by quantitative real-time PCR (qPCR) method to preliminarily explore the effect of SVV VP4 protein on cytokines, thereby providing an important basis for the pathogenic mechanism of the virus and vaccine design.

## 2 Materials and methods

### 2.1 Cells and viruses

The SVV CH-GDZQ-2018 strain (GenBank accession number: MN423333), 293 cells, pEGFP-C1 (+) vector containing the EGFP gene, and Top10 competent cells were preserved by our laboratory.

### 2.2 Primer design

Based on the full-length SVV gene sequence available in the NCBI GenBank database, primers for amplifying the SVV VP4 protein sequence were designed using Premier 5.0 software. The sequences were as follows: SVV-VP4-F: 5′-GGAAGATCTGGTAATGTTCAGACA-3′; SVV-VP4-R: 5′-CGCGGATCCTTTGAGGTAGCCAAGA-3′. The underlined regions in the forward and reverse primers represent the *Bgl II* and *BamH I* restriction sites, respectively. The expected amplification product was approximately 214 bp. The SVV VP4 gene-specific primers were synthesized by Nanjing GenScript Biotech Corporation.

### 2.3 VP4 gene amplification

Using reverse-transcribed SVV cDNA as the template, the VP4 gene was amplified with the SVV-VP4-F and SVV-VP4-R primers. The PCR reaction (50 μL) was performed under the following conditions: 95 C for 3 min (initial denaturation), 35 cycles of 95 °C for 15 s, 56 C for 15 s, 72 C for 30 s and final extension at 72 °C for 5 min. The PCR products were analyzed by 1% agarose gel electrophoresis. If the results matched expectations, the target gene was purified using a gel extraction kit (Juhemei Biotechnology Co. Ltd., Beijing, China).

### 2.4 VP4 product digestion and ligation

The VP4 PCR product and the pEGFP-C1 plasmid were digested with *BamH I* and *Bgl II* enzymes (Thermo Fisher Scientific, Shanghai, China). After digestion, electrophoresis was performed to confirm successful digestion. The purified VP4 gene was then ligated into the pEGFP-C1 expression vector.

### 2.5 Transformation and colony PCR screening

The ligation product was transformed into competent cells and plated on kanamycin-resistant Luria-Bertani (LB) agar plates, followed by overnight incubation at 37 C for 15 h. Three single colonies were randomly selected and inoculated into 700 μL of kanamycin-supplemented LB broth. After mixing, 1 μL of the bacterial suspension was used for colony PCR screening.

### 2.6 Plasmid digestion verification

Plasmid pEGFP-C1-VP4 was extracted from PCR-positive colonies and subjected to double digestion with *BamH I* and *Bgl II*. Successful digestion confirmed the correct plasmid construction. The positive plasmid was sent to Nanjing GenScript Biotech Corporation for sequencing.

### 2.7 Cell culture

A total of 293 cells preserved in liquid nitrogen were thawed rapidly in a 37 °C water bath and resuspended in DMEM medium (Thermo Fisher Scientific, Shanghai, China) supplemented with 10% fetal bovine serum (FBS) (Thermo Fisher Scientific, Shanghai, China) and 1% penicillin–streptomycin (P/S) (Solaibao Biotechnology Co. Ltd., Beijing, China). The cells were cultured in a 37 C, 5% CO_2_ incubator. When cell confluence reached 90%, they were passaged. After confirming good cell viability and sufficient density, the cells were seeded into 12-well plates for transfection.

### 2.8 Western blot analysis

When the cell density of the 12-well plate reached approximately 80%, the successfully constructed pEGFP-C1-VP4 plasmid was transfected into 293 cells by the liposome transfection method with the ExFect transfection reagent kit (Vazyme Biotechnology Co., Ltd., Nanjing, China). The cells were cultured at 37 C for 24 h, and a blank 293 cell control group was set up simultaneously. Cell proteins were collected 24 h after transfection for SDS-PAGE electrophoresis and transfer. GFP murine monoclonal antibody (1:1,000) (Cowin Biotech Co. Ltd., Beijing, China) was used as the primary antibody, and horseradish peroxidase-labeled sheep anti-mouse IgG (1:10,000) (Cowin Biotech Co. Ltd., Beijing, China) was used as the secondary antibody for the identification of VP4 protein.

### 2.9 qPCR for cytokine detection

A total of 293 cells were spread on 12-well plates and cultured in a 5% CO_2_ and 37 C incubator until the cell density reached approximately 80% for transfection. Experimental groups: pCMV-HA (blank plasmid) transfection group and recombinant plasmid pEGFP-C1-VP4 transfection group, with three replicates in each group. Transfection was set up in three time periods (12, 24, and 36 h). Cells were recovered, nucleic acids were extracted, reverse transcription was performed, and then qPCR detection of cytokines was carried out using the primers (Supplementary Table 1). Reaction procedure: pre-denaturation at 95°C for 30 s; 95 C for 5 s, 60 C for 35 s, a total of 40 cycles.

### 2.10 Statistical analysis

The qPCR data was analyzed using the 2^−ΔΔCt^ method for relative quantification. The results were visualized using GraphPad Prism 5.0 software. Statistical significance was denoted as follows: *(*p* < 0.05) represented statistically significant; **(*p* < 0.01) represented highly significant; ***(*p* < 0.001) represented extremely significant; (*p* > 0.05), ns represented no statistically significant.

## 3 Results

### 3.1 SVV VP4 expression plasmid construction and protein expression identification

**Figure 1:**
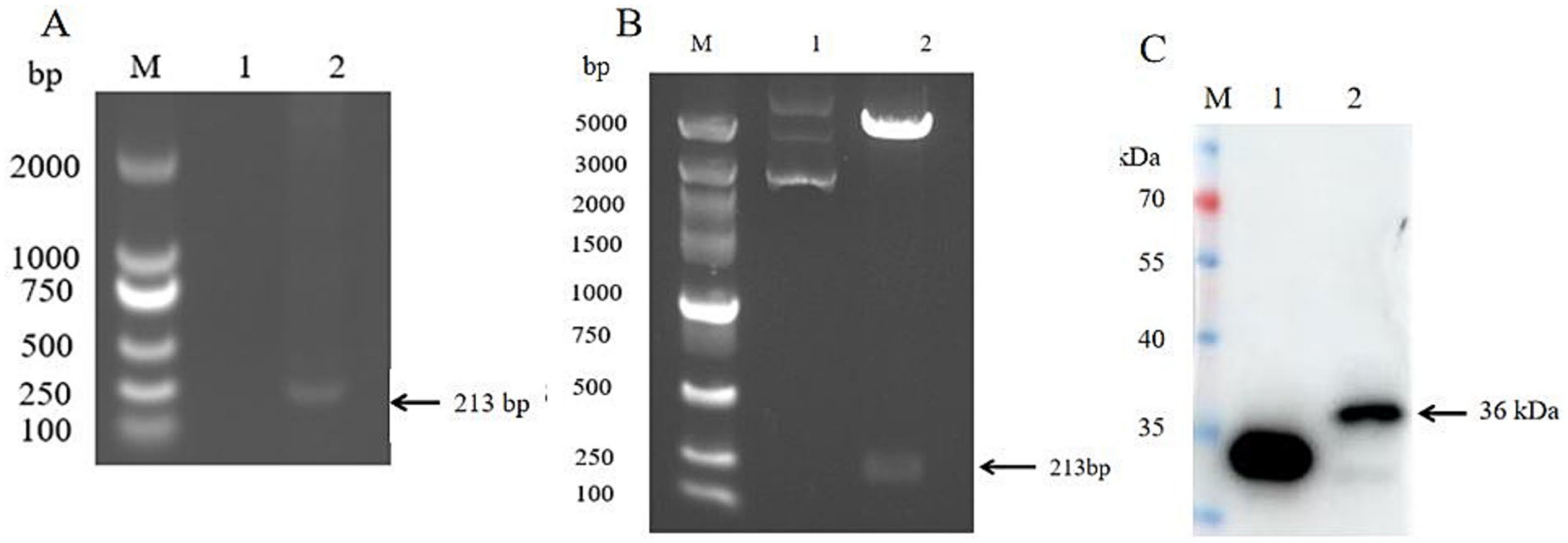
SVV VP4 expression plasmid construction and protein expression identification. **(A)** SVV VP4 gene amplification; lane1: negative, lane2: sample; **(B)** SVV VP4 expression plasmid double enzyme digestion identification; lane1: VP4-non-digested, lane2: VP4-digested; **(C)** SVV VP4 protein expression identification in vitro by Western blot. lane1: Control, lane2: VP4.

Using SVV nucleic acid as the template, RT-PCR was performed for amplification. The amplification product was subjected to 1% agarose gel electrophoresis. The result showed that a clear and specific band of approximately 213 bp, consistent with the expected fragment size. This result confirmed SVV VP4 gene was successfully amplificated (Figure 1A). Three colonies from the transformed agar plates resistant to kanamycin were randomly selected and inoculated into LB medium for cultivation. Subsequently, PCR amplification was performed. 1% agarose gel electrophoresis showed that all three colonies had specific bands. The bacterial solutions were sent to the company for sequencing. The bacterial solutions with correct sequences were used for plasmid extraction and were named pEGFP-C1-VP4. The recombinant plasmid was subjected to double enzyme digestion with *BamH I* and *BgI II* restriction enzymes. The 1% agarose gel electrophoresis results showed that a specific band of approximately 213 kb was cut out, which was consistent with the expectation, confirming that the recombinant plasmid pEGFP-C1-VP4 was successfully constructed (Figure 1B). Subsequently, the expression product of recombinant plasmid pEGFP-C1-VP4 was detected by SDS-PAGE and immunoblotting assay. The results showed that a clearly antigenic band appeared on the nitrocellulose membrane, with a molecular weight of approximately 36 kDa. This result confirmed that the expressed protein was the recombinant VP4 protein with an EGFP tag (Figure 1C).

### 3.2 The effect of overexpression of SVV VP4 on IL

**Figure 2:**
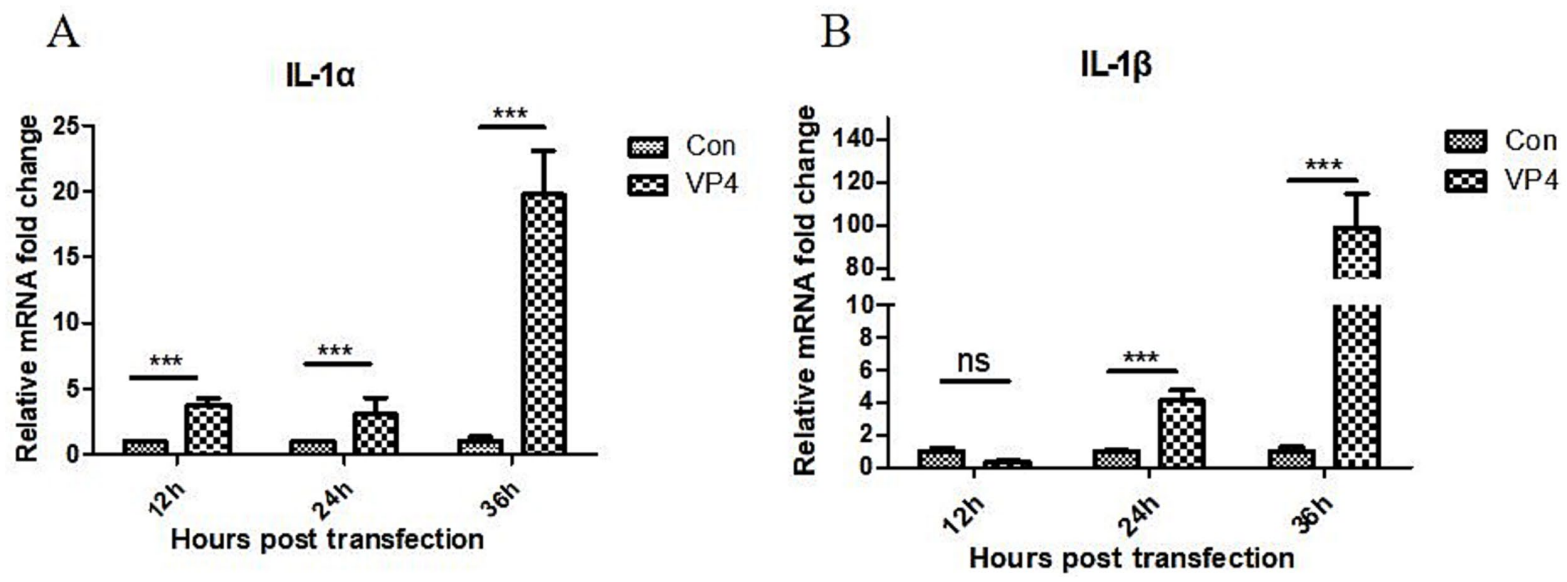
SVV VP4 overexpression regulates IL-1*α* **(A)** and IL-1β **(B)** expression. ***p < 0.001 represented extremely significant.

The pEGFP-C1-VP4 recombinant plasmid were transfected into 293 cells. Cells were collected at 12, 24, and 36 h after transfection, and the transcriptional level of IL-1α and IL-1β in the cells was detected by quantitative real-time PCR (qPCR). As shown in Figure 2, the VP4 protein could significantly promote the transcription of IL-1α at 12, 24, and 36 h, and promote the transcription of IL-1β only at 24 and 36 h.

### 3.3 The effect of overexpression of SVV VP4 on chemokines

**Figure 3:**
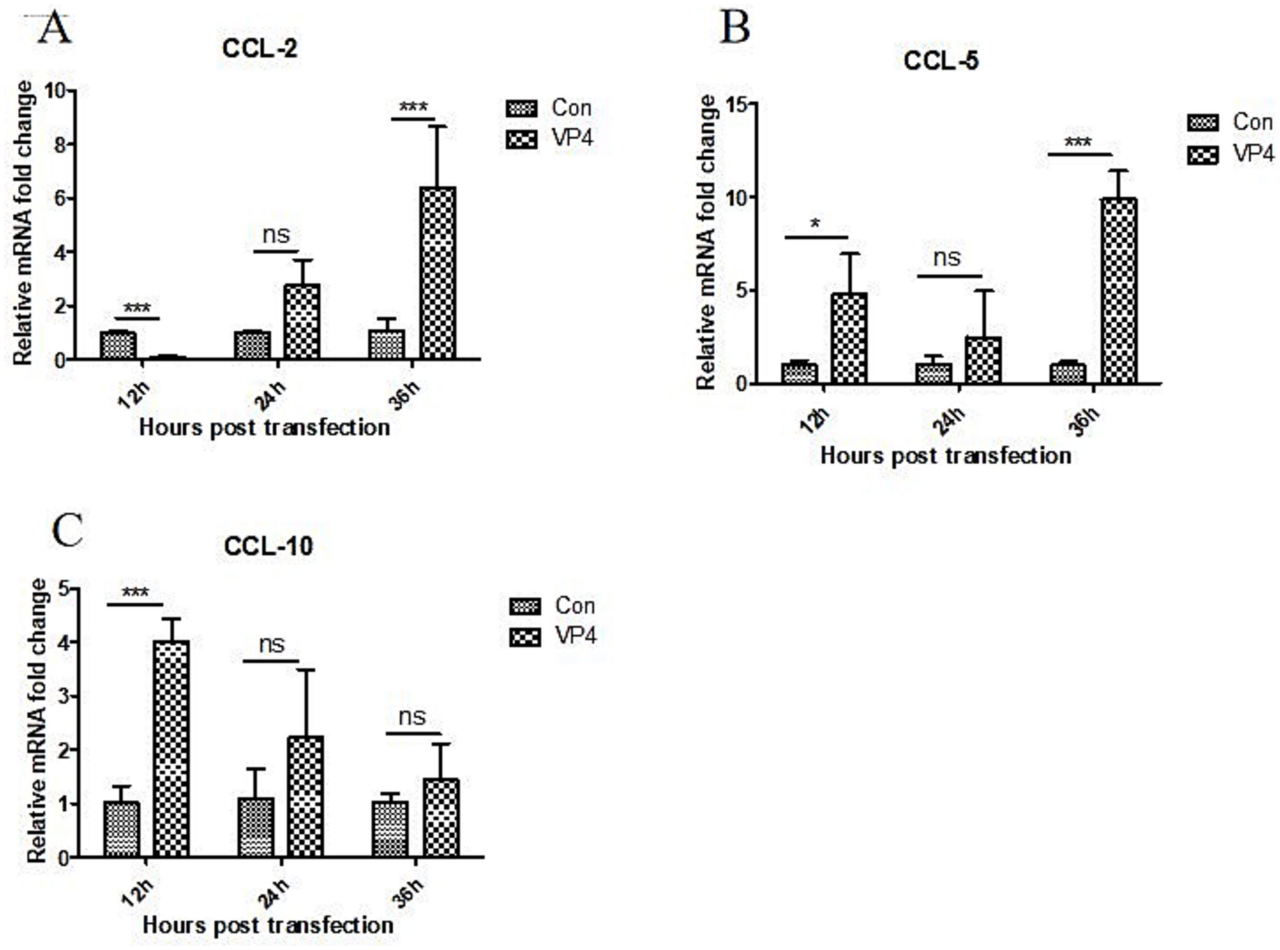
SVV VP4 overexpression regulates CCL-2 **(A)**, CCL-5 **(B)** and CXCL-10 **(C)** expression. *p < 0.05 represented statistically significant; ***p < 0.001 represented extremely significant.

After transfection, the transcriptional levels of CCL-2, CCL-5, and CXCL-10 in the cells were also detected by qPCR. As shown in Figure 3, the VP4 protein could significantly inhibit the transcription of CCL-2 at 12 h, and promote the transcription of CCL-2 at 36 h. However, there was no significant effect on the transcription of CCL-2 at 24 h. In addition, the VP4 protein could significantly promote the transcription of CCL5 at 12 and 36 h, and there was no significant effect on the transcription of CCL-5 at 24 h. We also found that the VP4 protein could significantly promote the transcription of CXCL-10 at 12 h, but there was no significant effect on the transcription of CCL-10 at 24 and 36 h.

### 3.4 The effect of overexpression of SVV VP4 on tumor necrosis factor

**Figure 4:**
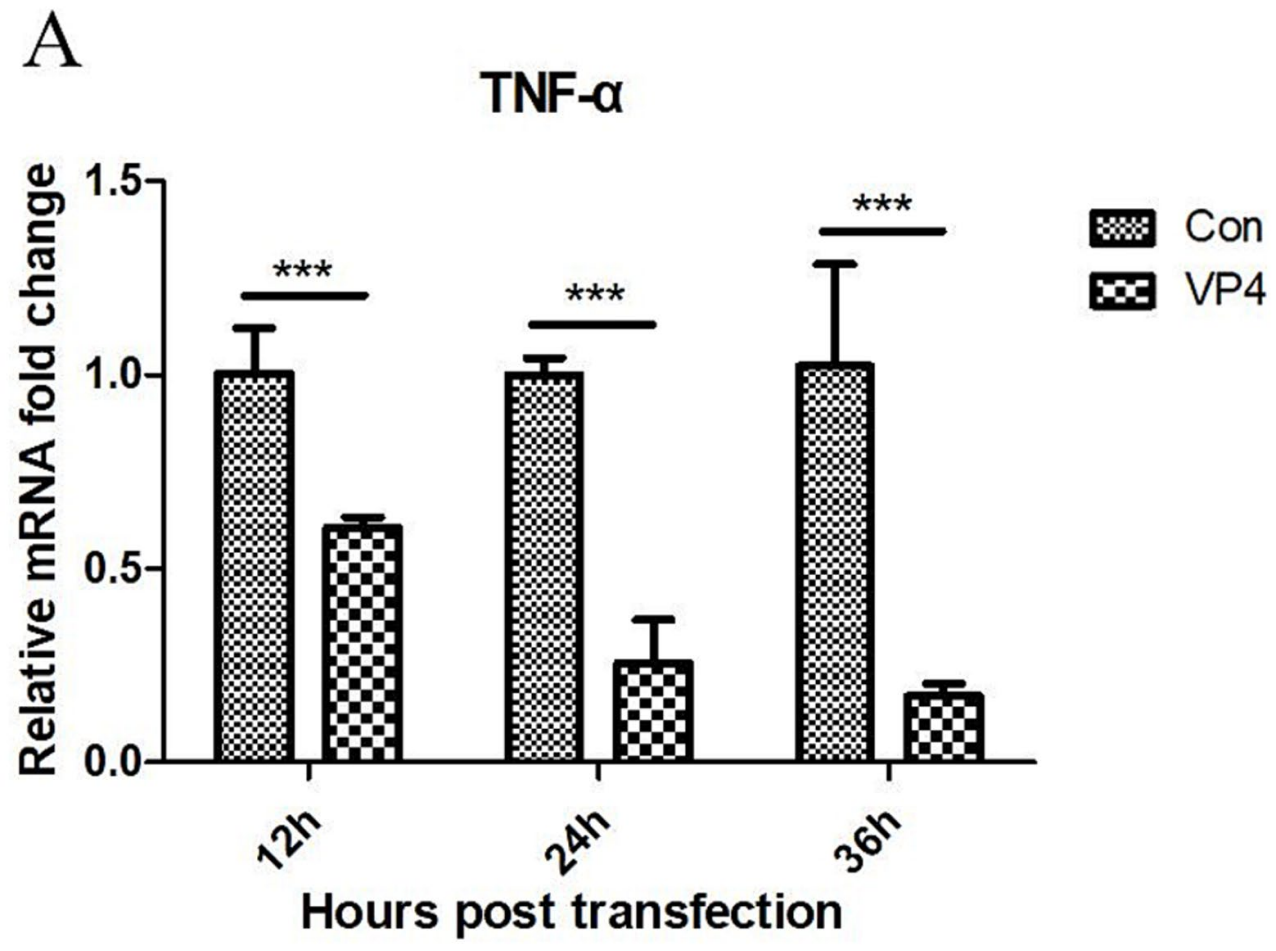
SVV VP4 overexpression regulates TNF-α expression. ***p < 0.001 represented extremely significant.

The transcription level of TNF-*α* in the cells was detected by qPCR after transfection. As shown in Figure 4, the VP4 protein could significantly inhibit the transcription of TNF-α at 12, 24, and 36 h.

## 4 Discussion

SVV disease is widely distributed in various regions of China. Since its first discovery in Guangdong province, many provinces have been successively reported SVV infection outbreaks (3–6). As it is an RNA virus with strong variability, the future epidemic situation and variant strains of this virus are difficult to predict, and currently there is no commercial vaccine (8). Cytokines play an important role in the innate immunity of the body. To explore the effect of SVV VP4 protein on cytokines and to provide theoretical basis for the formulation of antiviral strategies, vaccine design, disease diagnosis and treatment, the present study constructed a plasmid expressing the VP4 protein and transfected it into 293 cells *in vitro*. qPCR assay was carried out to observe the effects of VP4 transfection at different time points on 1 L-1*α*, IL-1β, CCL-2, CCL-5, CXCL-10, and TNF-α.

After the virus infects the host, the host itself often rapidly regulates the expression of cytokines and activates the local inflammatory response (14, 15). When African swine fever virus (ASFV) infects the host, it can result in exacerbated immune responses and increase levels of serum pro-inflammatory IL-1α and IL-1β (16). Rotavirus infection can also significantly increase the level of 1 L-1β in serum (17). Further study showed that ASFV H240R inhibited the NF-κB signaling causing a significant reduce in the transcription level of host 1 L-1β (18). Hepatitis B virus inhibits LPS-induced NLRP3 inflammasome activation and IL-1β production by suppressing the NF-κB pathway (19). These results indicate that activation of the NF-κB signaling pathway and caspase-1 are important steps in promoting the production of IL-1β. The results of this study show that the SVV VP4 protein can significantly promote the transcription of IL-1β. Whether the NF-κB pathway is involved in the regulation of IL-1β by VP4 requires further research.

During the inflammatory response process, the chemokines produced by the host can attract immune cells to the local tissue triggering an immune response and defending against pathogen invasion (15). Long coronavirus infection can upregulate CCL2 (20). The duality function of CXCL10, being either protective or pathogenic, is determined by the interplay between the host immune competence and the nature of the viral pathogen (21). Research has indicated a protective role for CXCL10 against specific RNA viral infections, including those caused by SARS-CoV and Epstein–Barr virus (EBV) (22, 23). Influenza A virus can induce CCL5 and CXCL10 level by TLR3 signaling pathway (24). Hepatitis A virus infection can increase the production of CXCL10 by RIG-I-like receptor signal molecules (25). This study shows that the SVV VP4 protein significantly inhibits the transcription of CCL-2 and promotes the transcription of CXCL-10 in the early stage. It can significantly promote the transcription of CCL-2 and CCL-5 in the later stage. These results indicate that VP4 can regulate the expression of chemokines. TNF-*α* plays a crucial role in viral replication. The host can regulate TNF-α production through the NF-κB and JAK/STAT pathways to inhibit the replication of swine fever virus (26). Human immunodeficiency virus (HIV) can affect its own proliferation through the TNF/TNFR pathway. HIV can encode proteins to interfere with the TNF-*α* signaling pathway of host cells, thereby inhibiting the function of TNF-*α* and facilitating its own proliferation (27, 28). Human cytomegalovirus (HCMV) and Epstein–Barr virus (EBV) can encode proteins that mimic host cell receptors, and these mimetic receptors may competitively bind to TNF-*α*, thereby preventing the binding of TNF-α to its true receptor and inhibiting the function of TNF-*α*, facilitating their own proliferation (29, 30). In this study, the SVV VP4 protein can significantly inhibit the transcription of TNF-α, suggesting that the VP4 protein may affect virus replication by regulating the production of TNF-α.

## 5 Conclusion

This study demonstrates that the expressed SVV VP4 protein exhibits good activity *in vitro*. Overexpression of the VP4 protein significantly promotes the transcription of IL-1α, IL-1β, CCL-2, CCL-5, and CCL-10, and inhibits the transcription of TNF-α at different time points. These results provide an important basis for the pathogenic mechanism of SVV and vaccine design in the future.

## Data Availability

The datasets presented in this study can be found in online repositories. The names of the repository/repositories and accession number(s) can be found in the article/Supplementary material.
