# Supplementary material for: Seneca valley virus VP4 protein regulates the transcription of different cytokines in vitro
Source: Front Vet Sci. 2025 Oct 16;12:1675546. doi: 10.3389/fvets.2025.1675546 (PMC12571611; doi:10.3389/fvets.2025.1675546)
Supplement: Supplementary file 1 [file Table_1.DOCX]

Supplementary Material

**Seneca valley virus VP4 protein can regulate the transcription of different cytokines in vitro**

Chaoliang Leng^1^, Yu Ge^2^, Mengfan Ruan^2^, Wenxiao Zhao^2^, Ximei Yang^2^, Sainan Gao^3^, Hongyue Zhai^1^, Dandan Li^1^, Dan Rao^2*^, Jianguo Dong^2*^

**Correspondence:** Corresponding Author: [raodan2007@126.com](mailto:raodan2007@126.com); [dongjianguo213@163.com](mailto:dongjianguo213@163.com)

**Supplementary Table 1.** Primers used in this study

| Genes | Primer sequences (5' to 3') |
| --- | --- |
| β-actin | F: AGAGCCTCGCCTTTGCCGATCC  R: CATGCCGGAGCCGTTGTCGAC |
| IL-1α | F: AATGACGCCCTCAATCAAAG  R: TGGGTATCTCAGGCATCTCC |
| IL-1β | F: AGGCACAAGGCACAACAGGCT  R: AACAACTGACGCGGCCTGCC |
| CCL-2 | F: GCCTCCAGCATGAAAGTCTC  R: AGGTGACTGGGGCATTGAT |
| CCL-5 | F: CTGCCTCCCCATATTCCTCG  R: CACACTTGGCGGTTCTTTCG |
| CCL-10 | F: CACCATGAATCAAACTGCGA |
|  | R: GCTGATGCAGGTACAGCGT |
| TNF-α | F: CCCTCTGGCCCAGGCAGTCA  R: ATGGGTGGAGGGGCAGCCTT |
